# “Putting people in charge of their own health and care?” Using meta‐narrative review and the example of online sexual health services to re‐think relationships between e‐health and agency

**DOI:** 10.1111/hex.12895

**Published:** 2019-05-03

**Authors:** Paula Baraitser, Alan Cribb

**Affiliations:** ^1^ King’s Centre for Global Health and Health Partnerships, School of Population Health and Environmental Sciences King’s College London, Weston Education Centre London UK; ^2^ School of Education, Communication and Society Waterloo Bridge Wing, Franklin Wilkins Building London UK

**Keywords:** agency, e‐health, sexual health

## Abstract

**Introduction:**

Policy discussions reference ideas of informed and active users of e‐health services who gain agency through self‐management, choice and care delivered outside clinical settings. In this article, we aim to problematize this association by “thinking with” material from multiple disciplines to generate higher order insights to inform service development, research and policy.

**Methods:**

Drawing on meta‐narrative review methods, we gathered perspectives from multiple disciplines using an iterative process of expert consultation to identify seminal papers citation mapping, synthesis and peer review.

**Results:**

We identify six relevant paradigms from sociology, philosophy, health services research, public health, the study of social movements and computer studies. Bringing these paradigms together illuminates the contrasting epistemological and ontological framings that co‐exist in this area, including competing conceptualizations of e‐health technologies as: neutral tools for service delivery, mediators within complex and unpredictable clinical interactions and as agents in their own right.

**Discussion:**

There is a need for e‐health policy to recognize many human and non‐human actors, the blurred boundaries between them and the unpredictable and evolving interactions that constitute engagement with e‐health care. Established models for e‐health service development and policy making are not designed for this landscape. There is nothing to be gained by asking whether e‐health, in general, either “increases” or “decreases” agency. Rather specific types and aspects of e‐health have diverse effects and can be simultaneously enabling and disempowering, and be differentially experienced by differently positioned and resourced actors.

## 1 INTRODUCTION

E‐health “the use of information and communications technologies in support of health”^1^ is associated with ideas about agency in health care. Policy discussions often assume that increased access to health information, new strategies for communication between health‐care users/providers and new tools for self‐management will change clinical relationships^2^ with predictions that e‐health will generate more informed users who more actively manage their health care.^3^ In this paper, we problematize these assumptions about e‐health and agency by deploying a meta‐narrative review approach in conjunction with consideration of the example of online sexual health services, thereby providing a richer policy‐relevant account of the possible relationships between e‐health and the agency of both users and providers of services. Our prompt to investigate these questions was the experience of a four‐year evaluation of e‐sexual health services,^4^, ^5^, ^6^, ^7^, ^8^ where agency emerged as an important construct in conversations from initial funding applications and through service development and delivery.^4^ Online sexual health services offer sexual health information, testing and treatment for sexually transmitted infections (STIs) with tests sent home, samples collected by the service user and posted to the laboratory and results sent by text message.^5^ In the United Kingdom, these are increasingly commissioned by the National Health Service and are free at the point of use. The case for the development of these services is strongly linked to ideas of engaged users actively managing their care by informing themselves, testing themselves and treating themselves within online services.^4^

In the United Kingdom, ideas about informed and active health‐care users feature strongly within policy discourses, with the right to be involved in planning and making health care‐related decisions set out in the Health and Social Care Act^9^ and the NHS Constitution.^10^ These documents specify the importance of shared decision making and choice in health care. Government policy links the discourse on the active health‐care user with predictions that e‐health will support this process; for example, NHS Digital, the national information and technology partner, aims to develop digital strategies that “put people in charge of their own health and care”.^11^ In this way, e‐health services are closely linked with ideas about agency, through self‐management, choice and the delivery of care within non‐clinical settings.

Despite its importance in policy discourse, the argument for a link between e‐health and agency is far from straightforward. For example, as well as providing opportunities for information and self‐care, e‐health services may constrain agency by requiring new skills and additional work from health service users, facilitate clinical intrusion into private spaces and reduce choice as face‐to‐face care is withdrawn.^2^, ^12^, ^13^, ^14^ E‐health services may similarly constrain the agency of clinicians, challenging their control of the process of care delivery through remote consultations or computer algorithms that make diagnoses and recommend care options.^15^

The proposed relationship between e‐health and active health service users and providers is complex and requires critical review. Whilst the notion of “agency” has emerged as central in the development of online sexual health services and as a useful construct through which to ask planning and evaluative questions, it is a problematic construct itself. Agency has specific meanings within different paradigms, and here, we rely on its simplest sense, that is, the “ability to act.” We are aware, for example, of its relation to ideas about human self‐determination from a range of contrasting perspectives, such as Christian theology, humanist philosophy and neoliberal political movements. Within some paradigms, agency has been assigned to some individuals, some animals and some actors and not others.^16^, ^17^ Within sociology, agency it is often used in relation to structures that might determine or limit the ability to act, and within philosophy, intentionality to act is an important factor. In bio‐medical discourses, agency may be used in reference to shared decision making in health care^19^ possibly with a requirement to engage even when engagement is not wanted.^20^ We have used the notion of “agency,” rather than the more specific idea of “empowerment” as the focus of this analysis—even though the latter is often used in health‐care research and policy—both because it is more descriptively and normatively open‐ended and because it is more commonly applied to non‐humans including digital technologies. For an analysis that focuses on e‐health care, this breadth seems important. The complexities surrounding the idea of agency point to the relevance of “standing back” from dominant health policy discourses and embracing broader lenses and perspectives in our exploration of the association between agency and e‐health care. In what follows we use a meta‐narrative review approach to “think with” material from a wide range of paradigms to generate higher order insights to inform service development, research and policy^21^ and to apply these to a current example to illustrate and develop the findings.

## 2 METHODS

In accordance with our aim to “think with” material from a wide range of paradigms to generate higher order insights on the relationship between e‐health and agency, we completed a meta‐narrative review, including illustrating and developing emerging insights with examples from online sexual health services. A meta‐narrative review considers a topic from multiple paradigms, collating ideas through a process of comparison across disciplines.^22^ E‐health technologies are complex interventions involving multiple actors with complex behaviours in open systems. Meta‐narrative review is one way to make sense of them by developing narratives that map relevant thinking across disciplines—developing new insights through comparing and contrasting approaches to the same topic.^21^ Teasing out the storylines of different research traditions and evaluating them in their own terms it asks: what different research traditions might be relevant to this issue? How is the topic conceptualized in each tradition? What are the key theories? What are the preferred study designs and ways of knowing? What are the main empirical findings? It incorporates six principles: *pragmatism*—include what makes most sense for the intended audience; *pluralism*—look at the topic from multiple perspectives; *historicity*—map changes in thinking within each discipline over time; *contestation*—conflicting thinking from different research traditions can generate higher order insights; *reflexivity*—reviewers should continually reflect on the emerging findings; peer review—emerging findings should be discussed with external audiences.^22^, ^23^ Meta‐narrative review is a two‐stage process. The first stage aims to map and summarize paradigms that offer relevant thinking, and the second is to compare and contrast these to generate higher order insights.

**Figure 1:**
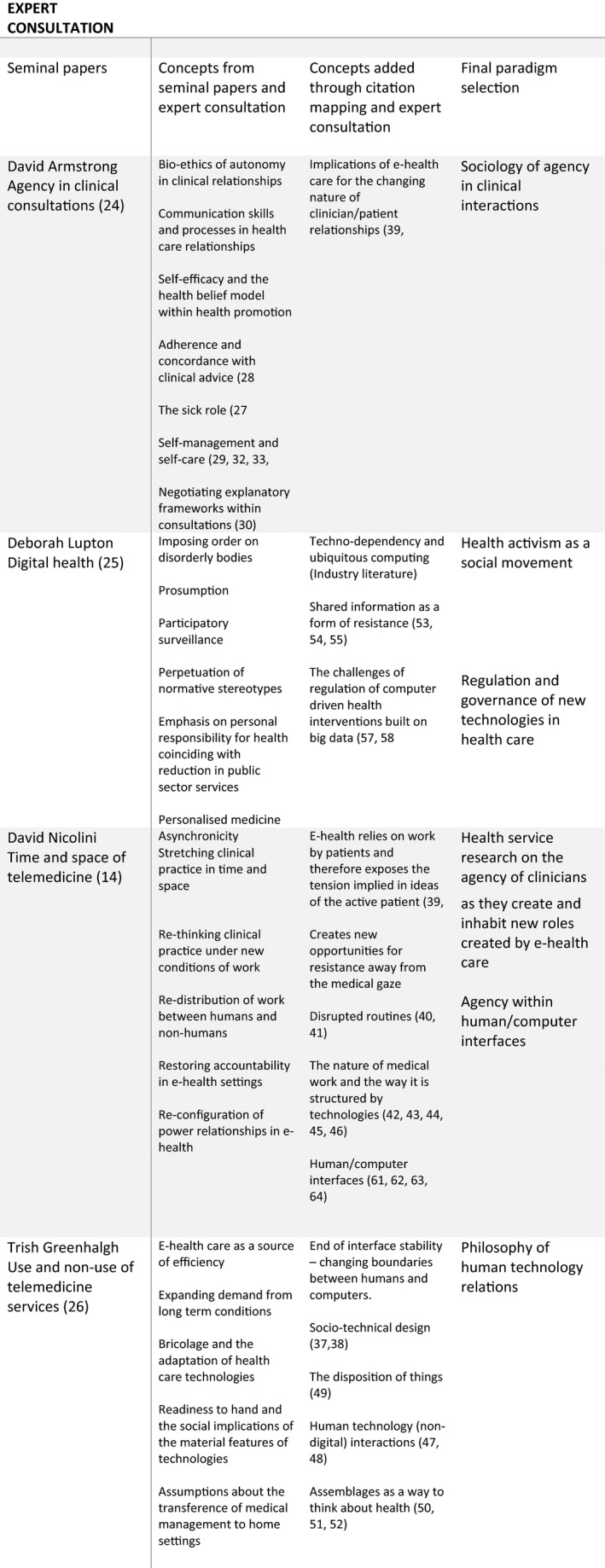
Process of paradigm selection

Our mapping phase is described in Figure 1 and was informed by a process of expert consultation within anthropology, sociology, applied philosophy, health‐care policy, health services research and e‐health services to identify seminal papers and their implications. The seminal papers that emerged were as follows: David Armstrong's work on agency^24^; Deborah Lupton's work on digital health^25^; David Nicolini's work on the time and space of telemedicine^14^; and Trish Greenhalgh's work on the use/non‐use of telehealth care.^26^ We used citation mapping and ongoing expert consultation to identify the concepts underpinning this work and to explore new ones. In each case, we evaluated research papers in terms of their ability to generate new thinking on the relationship between agency and e‐health care. We then summarized the results of our searches within six paradigms and generated a summary narrative within each.

During the synthesis phase, we built an over‐arching narrative to generate a rich picture of the topic from multiple perspectives and tested this through a process of peer review by individual specialists in sociology, improvement science, anthropology and two presentations of early thinking to academic audiences with discussion. This process generated a focussed comparative summary of different research traditions to generate new insights on the topic in question.^22^, ^23^

We identified and illustrated emerging higher order insights by considering the contribution of each paradigm to thinking about e‐health care through examples from our sexual health case study. The examples were informed by one author's (PB) experience of 5 years of development and evaluation of an online sexual health service. This process shows how a service can be re‐framed by “thinking with” each of the multiple paradigms we present and enables exploration of the implications of this approach.

## 3 RESULTS

First, we present six summary narratives that emerged as important for our analysis. In each case, their potential relevance for online sexual health services is used to help identify and illustrate insights (see Table 1). Following this, the narratives are collated with some points of connection and contrast indicated. The process of synthesis—especially the drawing out of tensions and higher order insights—is completed in the discussion section and illustrated with a summary of their implications for online sexual health services.

**Table 1:** Examples illustrating the relevance of the summary narratives to online sexual health services

Summary narrative	Example
1. The sociology of agency within clinician‐patient interactions and the impact of e‐health care on these.	1. Online contraceptive services seek to increase access to “the pill” by removing the need to engage with a clinical consultation. Users take on new responsibilities, inputting their medical histories and measuring and reporting their blood pressure. Clinicians develop new versions of clinical presence and relationships remotely including building trust, communicating risk, checking understanding and identifying and responding to inaccurate information. This may require communication through multiple media (text message, telephone) outside clinical spaces and normal opening hours. Users must decide whether and how to acquire the new skills required, manage related risk (eg deciding whether to report it accurately) and controlling what happens to their data.
2. Health services research on the impact of e‐health care and the agency of clinicians within health systems	2. Pre‐exposure Prophylaxis for HIV (PReP) is taken before sex that might pose a risk of HIV infection. The lack of public funding for PrEP in the UK engaged the historically important HIV advocacy community whose activism has tempered as HIV treatments improved. PrEP activists set up systems to privately purchase medication online forming new alliances with clinicians who provide monitoring and support but are not funded to treat. Clinicians adopted new roles in response—advocating for PrEP and adapting their services to provide monitoring and support. The fact that people can purchase generic medications from outside the UK online, but health‐care organizations cannot, created new clinical relationships in which clinicians were not the gatekeepers for medication.
3. Philosophy of human‐technology relations.	3. A hypothetical user, travelling home from work on the bus who receives her positive sexual health test from the online service by text message, can be described with reference to a combination of actors that interact in a specific time and place including: the settings on her phone that specify how much of the text message is visible immediately; the phone itself including properties such as battery life; the ability of those sitting close by to see the message; her predictions of their response; her experience of the infection as potentially stigmatizing, the information provided online, whether there is a clinic on the way home that she can visit for treatment and the algorithm that offers her online help. In this narrative, the possibilities of, and her experiences of, her agency at this moment will be constructed from all of these elements.
4. Health activism as a social movement.	4. A self‐managed approach to sexual health testing is increasingly taken for granted, acceptable and may increase testing rates. However, policies of self‐management can create new dilemmas for services. When people were offered a choice between free online HIV tests—one using a self‐sampling method where they take their own blood test and send it to the laboratory for processing and one requiring self‐testing where the test is completed at home, two thirds chose self‐testing (ie a completely self‐managed testing process) but only 57% of them reported their result to the service providing the test. This seems to be a clear “advance” for self‐management, but also represents a potential risk for HIV surveillance.
5. Regulation and Governance of new technologies in health care**.**	5. The Quality Care Commission(CQC) in England is concerned with the verification of identity and the assessment of competence to complete online medical histories prior to online prescriptions, particularly in services, such as sexual health services where there was no existing offline relationship such as might be the case in general practice. Prompts for CQC inspectors visiting digital services include: “How does the provider protect against patients using multiple identities?” and “How does the provider determine the patient's location at the start of consultations.” Appropriate answers to these questions in sexual health services are far from obvious and are being debated as standards and guidelines are written.
6. Agency within human/computer interfaces	6. In online sexual health service development, the valuing of user experience in the testing and modification of early prototypes through continued cycles of “build, test, learn” has had positive impacts on the engagement with online sexual health testing. The “tone of voice” of each communication; the way text messages are displayed; the ability to move between different media for communication with clinicians all influence the emotional experience of engagement and communicate the values of the service (Howroyd, 2017). This is particularly important in a service which involves the exchange of sensitive information and where service access may be experienced as stigmatizing.

### 3.1 The sociology of agency within clinician‐patient interactions and the impact of e‐health care on these

This narrative is drawn from qualitative study of consultations reported within medical sociology and medical anthropology. Agency is increasingly referenced within research on clinical consultations from the 1950s^24^ with the potential of e‐health care to influence this referenced from the early 1980s.

Early analyses of clinician‐patient interactions in the sociology of health and illness sometimes indicated a helpless, technically incompetent patient whose emotional involvement clouded their decision‐making capacities.^27^ Increasing acknowledgement of the importance of self‐care and evidence of poor compliance with clinical advice^24^ were important in re‐framing consultations as “patient‐centred”,^28^ with patients as experts^29^ and consultations as negotiations.^30^ This shift was associated with the development of self‐management programmes that overtly value the knowledge that comes from living with a long‐term condition^29^ and references to shared decision making in policy documents such as the UK Health and Social Care Act.^9^ Despite this discourse, patient advocates have argued that the implementation of shared decision making in clinical practice has been slow^31^, ^32^ despite training and resources to support change.^33^ More foundationally, Foucauldian‐inspired readings of self‐management have highlighted the ways in which the agency of both patients and clinicians, rather than being understood in contrast to governance, can be harnessed as a form of governance.^25^, ^34^

Computers, visible in consultations from the early 1980s, generated three‐way conversations. This had effects on clinical discourses, for example increased “doctor‐centred” speech 35; the structure of consultations, for example reduced opportunities for direct observation or examination 36; the information used for clinical care, for example biometric data collected through self‐monitoring 25; and the time and space of clinical interactions, for example more frequent, shorter interactions outside clinical spaces.^14^ A review of the impact of e‐health care on clinician‐patient relationships concludes that the varied impacts may include the replacing or disturbing of clinician‐patient relationships, strengthening patient participation or demanding more intense or more frequent participation.^2^

The complexities and uncertainties entailed by re‐worked clinical‐patient relationships, including new forms of patient participation, can be seen in online sexual health services (see Table 1). Here, there is no neat “transfer” of agency from clinicians to patients, nor is there a frictionless and “tidy” partnership; rather everyone has to develop and apply new forms of agency and “gains” of agency for patients entail “costs.”

### 3.2 Health services research on e‐health care and the agency of clinicians

This narrative draws on mixed‐method studies within health services research on the development and implementation of e‐health care within health delivery organizations. The focus on the effect of e‐health on the agency of clinicians provides a counterpoint to the focus on the agency of service users. This literature describes human‐technology relations within organizations as a product of both linear, designed and predictable relationships as well as complex and emerging ones.^37^, ^38^ Clinicians as both developers and users of e‐health interventions may support or constrain implementation^39^, ^40^, ^41^ with clinical roles challenged by e‐health care that is potentially less messy and inconsistent.^41^ Technologies also construct professional experience,^42^ for example, through remote communication or monitoring devices.^39^ Theories of technology adoption map influences on uptake such as: perceived usefulness/ease of use^43^; the social capital that comes from adoption^44^, ^45^; and ability to influence implementation^45^ to predict clinician engagement as users or supporters of use by others.^46^ The sexual health example of online pre‐exposure prophylaxis shows how “unmanageable” policy developments led to emerging roles for clinicians that then could become the basis for a degree of clinical ownership and planning. As well as being designers and users of e‐health services, clinicians (and their roles and agency) are also shaped by, or “products” of, e‐health care with clear implications for online sexual health services (see Table 1, Example 2).

### 3.3 Philosophy of human‐technology relations

This narrative, drawing on the philosophy of technology with particular reference to agency, underscores the mutually constitutive nature of humans and technologies. It includes Heidegger's influential distinction between technologies as “ready to hand” when their usefulness for a task makes their presence and properties invisible and “present at hand” when technologies are seen as objects and can be examined in their own right along with their specific attributes and functionalities. It captures the role of humans in modifying, appropriating and combining technologies using them for purposes for which they were not designed^47^ and the role of technologies in modifying human behaviour in intended and unintended ways. For example, carrying a camera constrains activities because of the need to protect it^48^ and clinical decision aids are specifically designed to change professional behaviour in certain ways but also produce other effects.^42^, ^49^

Later twentieth‐century philosophers include a much wider range of actors in human‐technology relationships, breaking down technologies into their component algorithms, interfaces and structures and acknowledging the importance of the places, affects, identities and relationships that influence experience of interactions.^13^, ^50^, ^51^ They point to blurred boundaries between humans and technologies, as bodies are understood through technologies and technologies are given meaning by the way they interact with bodies.^52^ They also introduce ideas of networked and unstable relationships involving multiple actors to create a particular interaction that may not be repeated and where the same actor may have different impacts in different networks.^16^ The object of study then becomes the assemblage of objects, actors and processes that mediate an experience of e‐health care. This is in contrast to the emphasis on socially structured, and more fixed, patterns of interactions described in traditional accounts of health service user/provider consultations and the impact of technologies on them. Example 3 in Table 1 shows the application of this thinking to online sexual health services.

### 3.4 Health activism as a social movement

This narrative draws on the history of health activism for rights to information and technologies to support self‐care. Health activist groups have emerged in response to diverse issues including: rights to information^53^; access to new technologies^54^; and the recognition of specific diseases.^55^ This increases the recognition of the contribution that people make to their own health care and, for example, strengthens advocacy for shared decision making with people seen as experts in their own condition.^56^ The generation of a group of active, engaged e‐patients’ who monitor their own condition, adjust their treatments, are networked with each other, access their own medical records and online health information is one extension of this^32^ but those who do not have the inclination or skills to actively manage their own health may be disadvantaged.^25^

In this context, e‐patients can be advocates for participatory medicine where “patients become potent agents in creating and managing their own health in partnership with physicians”.^56^ Such developments can create both opportunities and dilemmas for services (see Example 4, Table 1). Prominent e‐patients like e‐patient Dave (www.epatientdave.com) or Matt Eagles (www.parkinsonsmovement.com/project/matt-eagles/matt/) use e‐health communication strategies intensively to connect people with similar conditions, creating new repositories of information and discussion.

### 3.5 Regulation and Governance of new technologies in health care

Here, the emphasis is on the safety of medical devices requiring a structured approach to the introduction of new technologies from development to routine use. The safety of new technologies is maintained through systematic reviews of the literature, clinical trials, regulation of use, surveillance for unforeseen impacts and controlled access.^57^ Technologies are largely treated as discrete entities with predictable outcomes.

Given that e‐health care involves new kinds of technologies—less discrete and predicable—regulatory bodies acknowledge that new regulatory models and processes will be required and there is a commitment to developing these. They may include the regulation of complex “black box” algorithms that manage health‐care decisions outside the agency of both clinicians and service users^15^; artificial intelligence technologies that include some elements of unpredictability; and a need to ensure that the data used to drive these systems are legitimately accessed, robust and non‐discriminatory.^58^ In a sense, the whole point of emerging e‐health technologies is that they function (or “exercise agency”) in complex and unpredictable ways and this raises profound questions about the capacity to regulate them, especially by using established templates (see Table 1, Example 5).

### 3.6 Agency within human/computer interfaces

The impact of poor human/computer interfaces was highlighted in the 1970s and early 1980s when it became apparent that systems that were considered to be functionally excellent by computer scientists performed badly in the real world, generating stressed users, poor performance and decreased job satisfaction.^59^ Early research to address this focused on the user at a desktop, primarily in an office setting, performing well‐defined tasks. It drew on methodologies from engineering and psychology to study barriers to task completion that came from suboptimal human/computer communication.^60^ Subsequent research broadened its focus to include group working and computer‐mediated social interaction with a blurring of boundaries between home and work, between work and non‐work and between human and computer. This “second wave” of research acknowledged the agency of people as users of computers, the variability of their responses and the unplanned and responsive nature of most work.^61^ It refers specifically to the situated nature of human/computer interaction^62^ and is associated with a more participatory approach to design. A “third wave” of research looks at the interpretation and construction of meaning and emotion in human‐computer interaction, the importance of non‐task‐orientated computer use,^60^ the responsiveness of computers to their environments (such as phones that know their location), the presence of computers everywhere in the Internet of things and machine learning.^63^, ^64^ This paradigm overtly addresses the agency of non‐human actors within e‐health care. In this context, any residual associations that equate digital technology with “reasoning machines” need to be problematized and opened up given that some of the key dimensions of human actors—for example “style” or “character”—are relevant to technologies as they are experienced (see Table 1, Example 6).

### 3.7 Combining the narratives and applying them to e‐health policy and practice

The process of assembling and drawing together these six short narratives highlights the extent to which different currents of research, despite substantial overlaps, are built around different framings. In particular, different currents tend to construct actors and interactions differently and place emphasis on different sets of actors and interactions. Even a simple map of these ontological and epistemological divergencies indicates the diverse ways in which research has and might conceptualize the relationship between e‐health and agency. Here, in summary, we will highlight three contrasting, conceptualizations of the relationship.

First, e‐health technologies may be treated as tools to be used for specific interactions with planned and predictable outcomes which should be developed with evidence and monitored for safety. Related research might focus on the impacts of e‐health care on the agency of those who interact with them across populations and contexts and develop policy and regulatory frameworks that support clinical safety and effectiveness.

Second, e‐health technologies can be seen as mediators between service users and clinicians. Here, the focus is on the multifarious effects of technologies on: the time, space and content of interactions; the media they utilize; the conversations they generate, the values they reflect, the emotions they engender, and the way that they distribute the work required to become or remain healthy. In this conceptualization, technologies can be seen as having some “agency” but are largely seen as interfaces between the human actors (service providers and users) who remain the focus of the enquiry. This means that dyadic (human/health professional) or triadic (human/computer/health professional) are significant areas for research that looks at access, usability and clinical outcomes.

Third, e‐health technologies can be seen as non‐human actors (mobile phones, texts messages, user interfaces, algorithms) with their own agency contributing to networks that are transient and unpredictable. In this analysis, which reflects “new materialist” thinking, the distinctions between different types of actors, for example, humans and their condoms, or algorithms and the clinicians who wrote them, are less distinct than in the other two conceptualizations. In this “post‐human” narrative research shifts from individuals or technologies and aims to track the flow of assemblages.

Applying this thinking to digital technologies in sexual health care helps to identify and illustrate how these different currents of thinking construct different questions for policy makers and providers. E‐sexual health care within the first conceptualization is a channel shift of work from clinic to online that empowers users to take control of their health. Randomized controlled trial evidence supports its impact on uptake of testing,^5^ and online testing is a tool to reduce infections. The questions it raises are about supporting and resourcing channel shift, managing demand and understanding how service users move between online/offline modalities.

The second conceptualization of the e‐health care/agency relationship within online sexual health services focuses on technologies as mediators for human relationships including the recognition of the affective impacts of online interfaces. It acknowledges that computer algorithms and interfaces may be as “judgemental” as clinic staff and that online systems may be as rigid as clinic opening times for those trying to access care. It seeks to understand how complex conversations previously enacted face‐to‐face, such as screening for child sexual abuse, are conducted online.^65^ It questions the changing role of clinicians, additional work for users and “intervention‐generated inequalities” arising from e‐health services that are more accessible/acceptable/effective in specific populations.^66^ This work has implications for policy makers, clinical education and training as new clinical roles and services are required.

The third conceptualization acknowledges the complexity of assemblages within e‐sexual health services. It points to the time, place and context‐specific experience of an episode of e‐sexual health service care. It offers opportunities to think about the online interface, its language, the person using it, their partner, the algorithm that sits behind it, the marketing that presents it and the change in national regulations that support or constrain it. Here, we would anticipate learning from and building on the way technologies are modified and developed by users gaining new ideas from their creativity, expecting the same technology to become a different tool in different contexts and breaking it down into the multiple actors that come together for it to function. This conceptualization blurs boundaries between people and their phones or their software‐predicted next menstruation and the hormonal changes that “deliver” it.

If policy thinking is going to do justice to the richness of e‐health, it must be informed by, and be capable of moving between and across, such contrasting conceptualizations. Doing so entails coming to terms with layers of complexity and axes of contestability. E‐health does not redistribute agency according to “zero‐sum” rules nor in ways that can be neatly planned or managed by policy makers or clinicians; rather many such changes are unpredictable and emerge from and create new assemblages and forms of agency. In addition, emerging landscapes are normatively as well as descriptively complex. For example, health systems rightly strive to foster and respond to individual and collective patient agency but policy makers may need to balance this responsiveness with other public goods. Models of planning or evaluation need to be systematic and rigorously evidence‐based but if they are purely “technicist”—failing to encompass the aesthetic, affective and ethical aspects of e‐health technologies—they will not be equal to this task.

## 4 DISCUSSION

A meta‐narrative review approach, by highlighting pluralities and contestations, has the potential to act as a substantial stimulus to thinking about policy and service developments. We would suggest that this approach—which encourages a high level of intellectual reflexivity—is a useful complement to making progress within specific paradigms, because it supports and encourages scholarly “gestalt switches” that reorient agendas. For example, in this case, the approach helps dislodge any background assumption—sometimes embedded in health policy discourses—that e‐health is simply about “tools” that enhance the agency of clinicians and empower patients. More importantly, it indicates the range of intellectual resources needed to do justice to the topic. Opening things up in this way, almost by definition, does not provide “easy answers” to policy or practice problems, but it provides a more expansive set of possible “ingredients” for imagining ways forward.

The limitations of our approach and a challenge within this methodology in general are the requirement to limit the selection of paradigms to make synthesis possible and the necessarily succinct summaries of paradigms that inevitably lack detail and risk superficiality. During the iterative process of selection of paradigms, our core concern was to elicit breadth of perspective rather than attempt an in‐depth search within any single domain, but we made difficult decisions to exclude some paradigms, for example, the study of agency within economics or some elements within other paradigms, for example, ideas about “the quantified self” in health activism as a social movement.

Long established and embedded models are not designed for the shifting landscape represented here. For example, existing public health agency structures for the regulation of access to medical technologies seem unsuited for a world of expanding access to information and technologies for self‐care made possible by e‐health services. At a more fundamental level, long‐standing assumptions about the nature and locus of agency can be destabilized. Within some scholarly currents, agency is routinely ascribed to non‐human actors, and this has obvious relevance to any analysis of agency in e‐health care where technologies delineate what is possible, engineering specific clinical pathways and creating new possible identities for users.^67^ This broader ascription of agency is a powerfully generative move for re‐conceptualizing questions about the relationship between e‐health and agency (even for those who ultimately wish to resist this broader reading of agency). For example, it draws attention to the way that not only human beings but also computers (or other facets of ICT) can embody and reproduce purposes, values and emotions.

E‐health services have changed relationships in clinical consultations from intermittent, synchronous and often intense face‐to‐face consultations to interactions that are: asynchronous in time and space; delivered through remote media; include user generated data; introduce self‐management technologies; expand clinical interactions into home settings; and expect active participation by users**.** The assumed increase in agency associated with this development is partly dependent on the move out of the clinical space and away from the clinical gaze and the buffering of clinical relationships through digital media. However, it has also meant the intrusion of health services into new environments where they may monitor and record private activities, generating new and personal data that could be used within health‐care delivery and beyond. In this way, e‐health services produce new iterations of important questions about respectful and trusting clinical relationships where decision‐making processes are negotiated and where there are clear limits on sharing of personal information. Much of the literature within health policy and practice has framed computers as neutral channels within these relationships. However, as just noted, given that computers increasingly interact with their environments, demonstrate intelligence, stimulate emotions and embody values they are no longer (and arguably never were) neutral channels but responsive, intelligent, value laden and emotive actors within clinical relationships.

Clinical and health policy discussions have been slow to acknowledge the agency of technologies, their affective impacts, the values they embody, the instability and blurred boundaries of their relationships with humans and other actors. Policy that incorporated these ideas will need to engage, for example, with the fact that the style and character of e‐health interfaces impact on clinicians’ abilities to include and exclude specific populations, potentially generate further inequalities in access, and produce new clinical roles that require an updated clinical curriculum. These are all clear examples of the policy importance of “non‐human agency” in e‐health services. They reveal new actors, forces and relationships that might be mobilized to promote and maintain health,^68^ and they suggest that substantial policy developments will be required in response to e‐health services. In addition, guidelines and education on clinical interactions might also benefit from the representation of technologies as actors and a greater acknowledgement of their role in consultations.

There is nothing to be gained simply by asking whether e‐health (in general) either “increases” or “decreases” the agency of patients or clinicians. Rather we need to consider the complex ways in which the agency of relevant human actors can be constructed and inflected by specific types and aspects of e‐health in ways that might be simultaneously enabling and disempowering, and which are also differentially experienced by differently positioned and resourced actors. E‐health services can produce new kinds of freedoms for patients, for example with more independent forms of access to services beyond clinical environments, but these will also represent new forms of intrusion and call for new forms of responsibility. A similar “both/and” analysis applies to clinicians—e‐health represents an opportunity for more, and more radical, technical innovation for clinicians but these same technologies can powerfully structure clinical experience and even (more or less) displace clinical roles.

This review and discussion suggests an approach to service development and evaluation that assumes the presence of many human and non‐human actors, blurs the boundaries between them, identifies their components and expects unpredictable and evolving interactions that will constitute the agency of each. It emphasizes the importance of research on “the thing side,” that is the technologies that structure experience of e‐health services^67^ and acknowledges the distributed nature of health decision making that goes beyond single consultations and includes many people and things.^69^ This analysis indicates that sustained interdisciplinary research is required to inform intelligent policy making, including research to map the agency of technologies within e‐health care and to identify the full range of their actions in a given context. It also suggests activities within e‐health services that might be helpful to broker relationships between the different actors involved. For health‐care users, this may include support to understand, modify, adapt and possibly reject technologies or their components. For clinicians, it may include encouragement of a similar expectations of negotiation with technologies and training to do this but also new clinical roles to support users to do the same.
